# Fly or fall?

**DOI:** 10.36834/cmej.70318

**Published:** 2020-07-15

**Authors:** My-An Tran

**Affiliations:** 1 University of Toronto, Ontario, Canada

**Figure:**
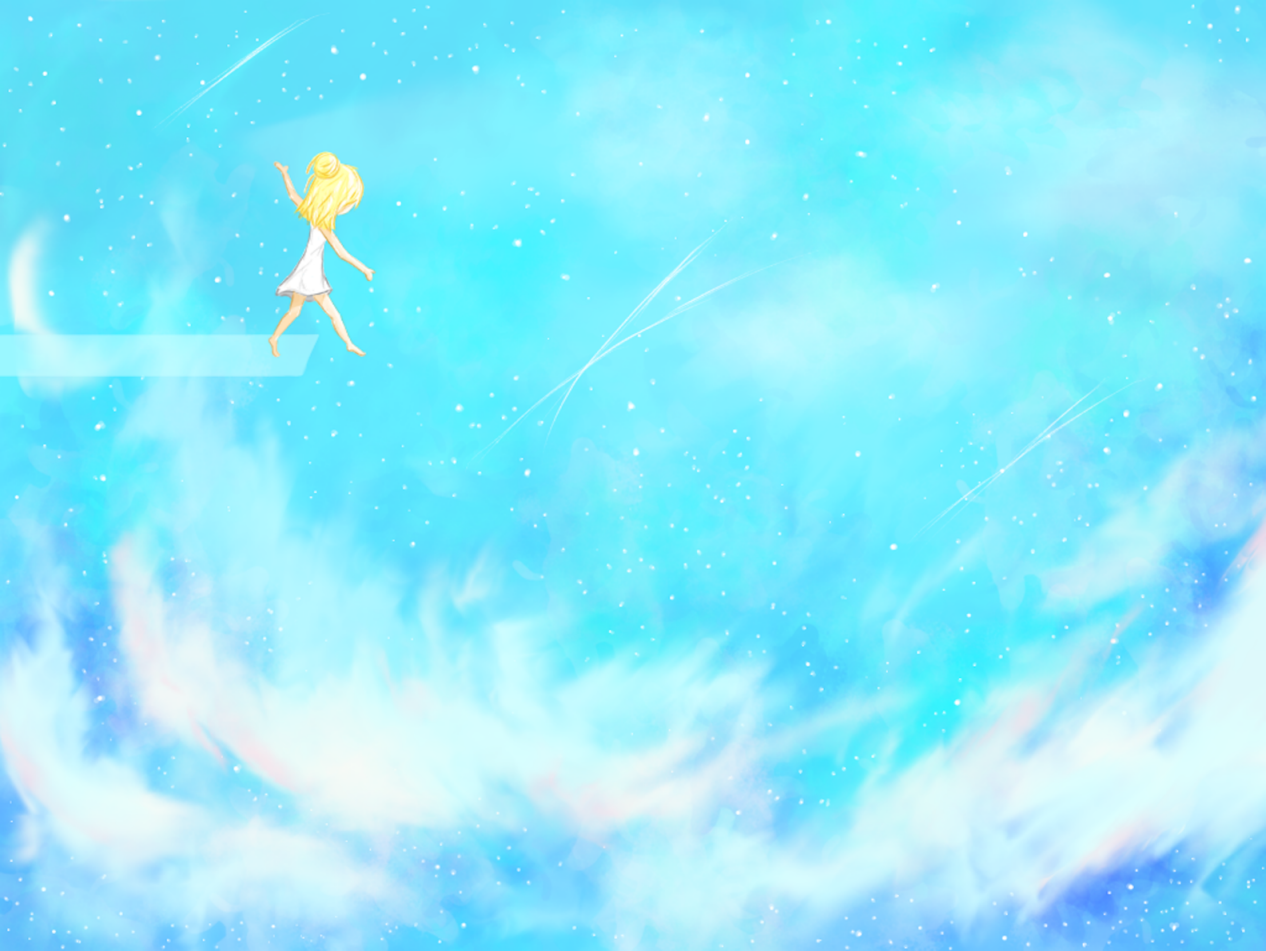


Being a medical student during the COVID-19 pandemic feels disorienting and disconcerting. But being an unmatched final-year medical student feels even more so.

During this year’s CaRMS cycle, I was one of eight in my class who did not match. I still clearly remember the whirlwind of emotions I felt when I found out. Initially, it started with disbelief. I was sitting in lunch rounds when I read the dreadful email, surrounded by residents and staff, and I remember not knowing what to do or how to feel. Everyone around me was discussing a case, the day was going on as usual, but I couldn’t hear a word anyone was saying anymore. Eventually, I got up from the table and left the room without a word, without an excuse. I found my way to a washroom and looked at myself in the mirror. That was when the tears finally came. That was the moment when disbelief turned into disappointment, despair, and a crushing sense of failure.

In the weeks to come, this sense of failure stayed with me, haunted me. It lingered in the shadows of my every thought. Fortunately, throughout those weeks I was met with unwavering support from family, friends, and faculty. With their help, we worked through the difficult decision-making that followed. Whether I should apply for second iteration, and if so for which programs; or whether I wait another year, and if so what would I do during that year—the options felt endless, overwhelming, and at times futile. Just as I was starting to gain a better sense of what I wanted to do, the COVID-19 pandemic struck and the plans that had started to solidify melted back into confusion. Timelines became uncertain. Opportunities were disappearing. I was lost again.

“Fly or fall” is a piece that captures this feeling, this uncertainty, as vast and as endless as the sky. The story I tried to impart in this digital painting is one that parallels my own: that of a girl who walks along a glass path that abruptly comes to an end. She knows that the next step she takes will send her tumbling through the sky and hesitates, wondering if she will learn to fly and successfully navigate the uncertainties that lie beyond the layer of clouds, or if she will fall and watch all the opportunities that fill the bright blue sky slip by as she plummets towards failure once again.

When I entered medical school, I thought my path was clear: one straight line from a bachelor’s degree to medical school followed by residency. This path had always been there to provide structure, and the comfort of knowing what comes next. But suddenly, this glass path disappeared from beneath me, and now I am no longer sure where my next step will take me, if the platform will hold me, or how I will forge a new route. There are so many things that I can choose to do, or choose not to do, during this pandemic and during its aftermath. But in the midst of this novel situation that we have never faced before, it is hard to anticipate what impact the choices we make will have on our futures, on our residency applications. As I walk on ahead, at the end of it all—the end of this pandemic, the end of this year’s CaRMS cycle—will I fly or will I fall?

